# Supplementary material for: Disentangling metabolic functions of bacteria in the honey bee gut
Source: PLoS Biol. 2017 Dec 12;15(12):e2003467. doi: 10.1371/journal.pbio.2003467 (PMC5726620; doi:10.1371/journal.pbio.2003467)
Supplement: S8 Data — (ZIP) [file pbio.2003467.s008.zip › Z-score_plots/522 microbial substrate trans-2-[(Dimethylamino)methylimino]-5-[2-(5-nitro-2- furyl)-vinyl]-1,3,4-o....pdf]

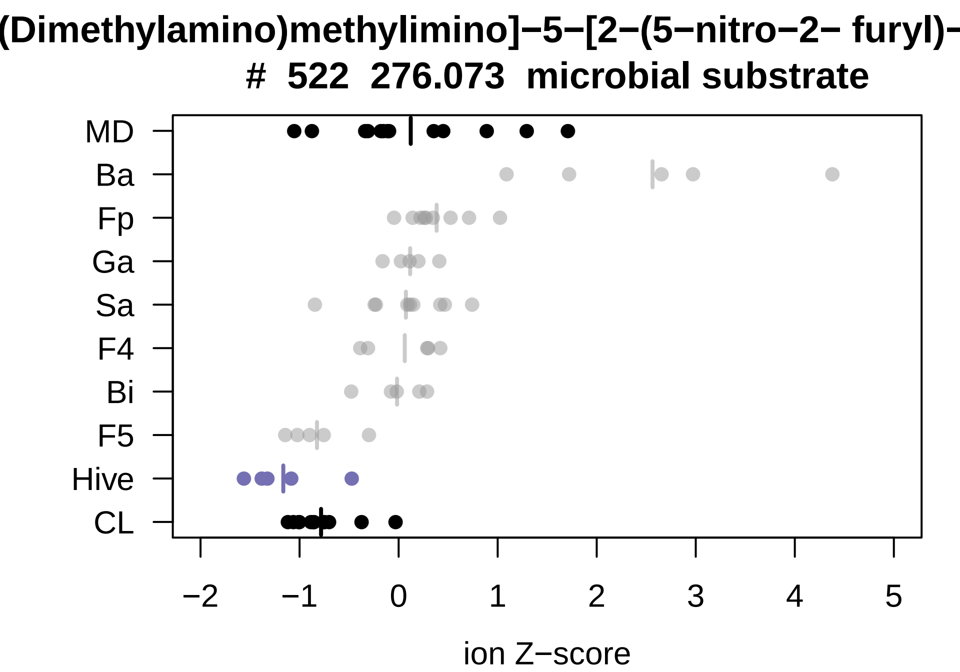

(Dimethylamino)methylimino]−5−[2−(5−nitro−2− furyl)−
522 276.073 microbial substrate
MD
Ba
Fp
Ga
Sa
F4
Bi
F5
Hive
CL
−2
−1
0
1
2
3
4
5
ion Z−score
